# A double dissociation between action and perception in bimanual grasping: evidence from the Ponzo and the Wundt–Jastrow illusions

**DOI:** 10.1038/s41598-020-71734-z

**Published:** 2020-09-04

**Authors:** Aviad Ozana, Tzvi Ganel

**Affiliations:** Department of Psychology, Ben-Gurion University of the Negev, 8410500 Beer-Sheva, Israel

**Keywords:** Human behaviour, Perception, Consciousness, Motor control, Object vision

## Abstract

Research on visuomotor control suggests that visually guided actions toward objects rely on functionally distinct computations with respect to perception. For example, a double dissociation between grasping and between perceptual estimates was reported in previous experiments that pit real against illusory object size differences in the context of the Ponzo illusion. While most previous research on the relation between action and perception focused on one-handed grasping, everyday visuomotor interactions also entail the simultaneous use of both hands to grasp objects that are larger in size. Here, we examined whether this double dissociation extends to bimanual movement control. In Experiment 1, participants were presented with different-sized objects embedded in the Ponzo Illusion. In Experiment 2, we tested whether the dissociation between perception and action extends to a different illusion, the Wundt–Jastrow illusion, which has not been previously used in grasping experiments. In both experiments, bimanual grasping trajectories reflected the differences in physical size between the objects; At the same time, perceptual estimates reflected the differences in illusory size between the objects. These results suggest that the double dissociation between action and perception generalizes to bimanual movement control. Unlike conscious perception, bimanual grasping movements are tuned to real-world metrics, and can potentially resist irrelevant information on relative size and depth.

## Introduction

The idea that the visual system is segregated to two functionally distinct visual pathways, a ventral stream, which mediates conscious perception, and a dorsal stream, which mediates guided actions, is supported by converging evidence from neurological and behavioral studies^1–3^.

In healthy subjects, one of the best-known (but also contentious) line of evidence for functional separation between action and perception comes from research on the effects of size-contrast illusions on precision grasping. A large number of studies reported that illusory-induced effects have a limited influence on the way people shape their fingers prior to grasp^4–9^. These findings were interpreted in light of the two visual systems account. It was suggested that guided actions, subserved by the dorsal stream, can operate independently from conscious perception and rely on dissociable computations of object shape and size^4,6^. However, other studies reported conflicting results using different measures and designs and offered alternative explanations for the original findings^10–13^ (but see^14^). Indeed, previous research showed that for some illusions such as the Ebbinghaus illusion, findings of functional separation between perception and action are not consistent across different methods and designs (for a review see^15^, also see the current debate in^16,17^).However, for other illusions, such as the Ponzo illusion, the functional dissociation between action and perception has been consistently replicated across different studies and labs^9,18–21^ (but see^21^, that reported small correlation effects between the influence of the illusion on perception and on grasping).

Previous research on the relation between action and perception mainly focused on one-handed, unimanual grasping. Nevertheless, in everyday life, people also interact with objects using both their hands. For example, bimanual grasping is commonly used to grasp objects of larger size, beyond the capacity of single-handed grasping. It is unclear, however, whether bimanual grasping could resist the effect of visual illusions. The motor control of bimanual grasping is associated with right hemisphere dominance^22–24^. However, efficient visuomotor control which subserves right-hand precision grasping has been linked to the involvement of the left hemisphere^19,25^. For example, Gonzalez et al.^19^ reported that grasping trajectories with the left, but not the right hand, are susceptible to size illusions. A different study conducted on patients with unilateral brain damage reported that patients with lesions to the left-hemisphere are more likely to be affected by the Müller-Lyer illusion during grasping. Conversely, patients with right-hemispheric damage were refractory to the illusion^26^. Furthermore, imaging and neuropsychological studies indicate a left hemisphere specialization for processing attributes related to fine visuomotor control (such as praxis selection), and a right hemisphere specialization for visuospatial processing^27–29^. These findings suggest that bimanual grasps may be more susceptible to contextual effects and to perceptual distortions. Then again, bimanual grasping is a commonly used type of movement that could arguably require a minimum amount of top-down supervision. It has been suggested that such well-practiced and automatic movements are less prone to potential effects of irrelevant aspects of the object and its surroundings^19,30^.

Only scarce research examined the relation between action and perception in bimanual grasping. In a recent study from our lab^31^, bimanual grasping and perceptual estimations were tested for their adherence to Weber’s law. The results showed that just as in unimanual grasping, bimanual grasping (but not bimanual perceptual estimates) violated Weber’s law. These results suggest that bimanual and unimanual grasping rely on a shared mechanism that encodes target size in an analytic rather than relative fashion. Yet, the above study focused on the visual resolution of the response, while susceptibility to size-contrast illusions reflects context-dependent processing that biases perception.

To the best of our knowledge, unlike in the case of unimanual grasping, only a few previous studies looked at bimanual grasping trajectories in the context of visual illusions. In these studies, participants were asked to simultaneously grasp two (small) same-sized objects, one in each hand. The results were inconsistent; For example, while Dewar and Carey^32^ showed that bimanual grasping trajectories are immune to the Müller–Lyer illusion, a follow-up study by Foster et al.^33^ showed illusory effects on bimanual grasping, but only when visual feedback was removed upon grasp. In an earlier study by Vishton and Fabre^30^, bimanual grasping movements were refractory to the Ebbinghaus illusion in easy, but not in difficult lifting conditions. We note that previous studies in this domain were limited in two aspects; First, target objects in these studies were of relatively small sizes, which are less likely to be grasped with both hands in everyday situations. In addition, previous studies did not pit real against illusory size in their designs^18^. Therefore, these studies did not compare the effects of physical and illusory size differences on action and perception.

The current study was aimed to overcome these issues by using objects of larger sizes, that can be more naturally grasped by both hands^31^ and by applying a design that pits real against illusory size in bimanual grasping^18^. In this paradigm, two objects of different sizes are embedded in a size-contrast illusion (e.g., the Ponzo illusion in Experiment 1). The relatively small differences between the physical sizes of the objects are contrasted by the illusory context which induces an opposite perceptual effect. In such situations, participants often erroneously perceive the larger object in the pair as the smaller one (and vice-versa). Yet, previous experiments showed that in unimanual grasping, the aperture between the fingers reflects the real-not the illusory-size differences between the objects^18^. This double dissociation between perception and action in the context of illusions provides compelling evidence for the idea that perception and action compute different aspects of the visual environment. However, such dissociation has been documented only for unimanual grasps. Would the double dissociation between action and perception extend to bimanual grasping?

In Experiment 1, we asked participants to grasp large objects embedded in the Ponzo illusion^9,18–20^ using both their hands. In Experiment 2, we tested whether the results would generalize to a different visual illusion, that was not previously used in grasping experiments—the Wundt–Jastrow Illusion.

## Experiment 1

### Methods

#### Participants

Twenty-eight healthy psychology students were equally allocated to one of two conditions. The bimanual grasping group (Experiment 1a, 4 males, average age = 24.7, SD = 2.9), and the bimanual perceptual estimations group (Experiment 1b, 5 males, average age = 25.1, SD = 1.4). The participants provided informed consent and received monetary compensation for their participation (the equivalent of 10$ for the longer grasping condition or 5$ for the shorter manual estimations condition). The results of one participant from the bimanual perceptual estimation group, who did not follow the experimental instructions, were removed from the analysis.

The experimental protocols in experiments 1 and 2 were approved by the Human Subjects Research Committee at Ben-Gurion University of the Negev (submission #1257). The study adhered to the ethical standards of the Declaration of Helsinki. All participants signed a consent form prior to their participation in the experiment. The manuscript contains no information or images that could lead to identification of a study participant.

#### Apparatus and stimuli

The participants’ grip scaling was tracked using an Optotrak Certus device (Northern Digital, Waterloo, ON). The apparatus tracked the 3D position of two active infra-red light-emitting diodes attached separately to the participant’s left and right index fingers (200 Hz sampling rate). We used Computer-controlled PLATO goggles (Translucent Technologies, Toronto, ON) with liquid–crystal shutter to control stimulus exposure time, and a custom-written MATLAB code to control trial sequences and events (version 9.4, The Mathworks, Natick, MA).

**Figure 1:**
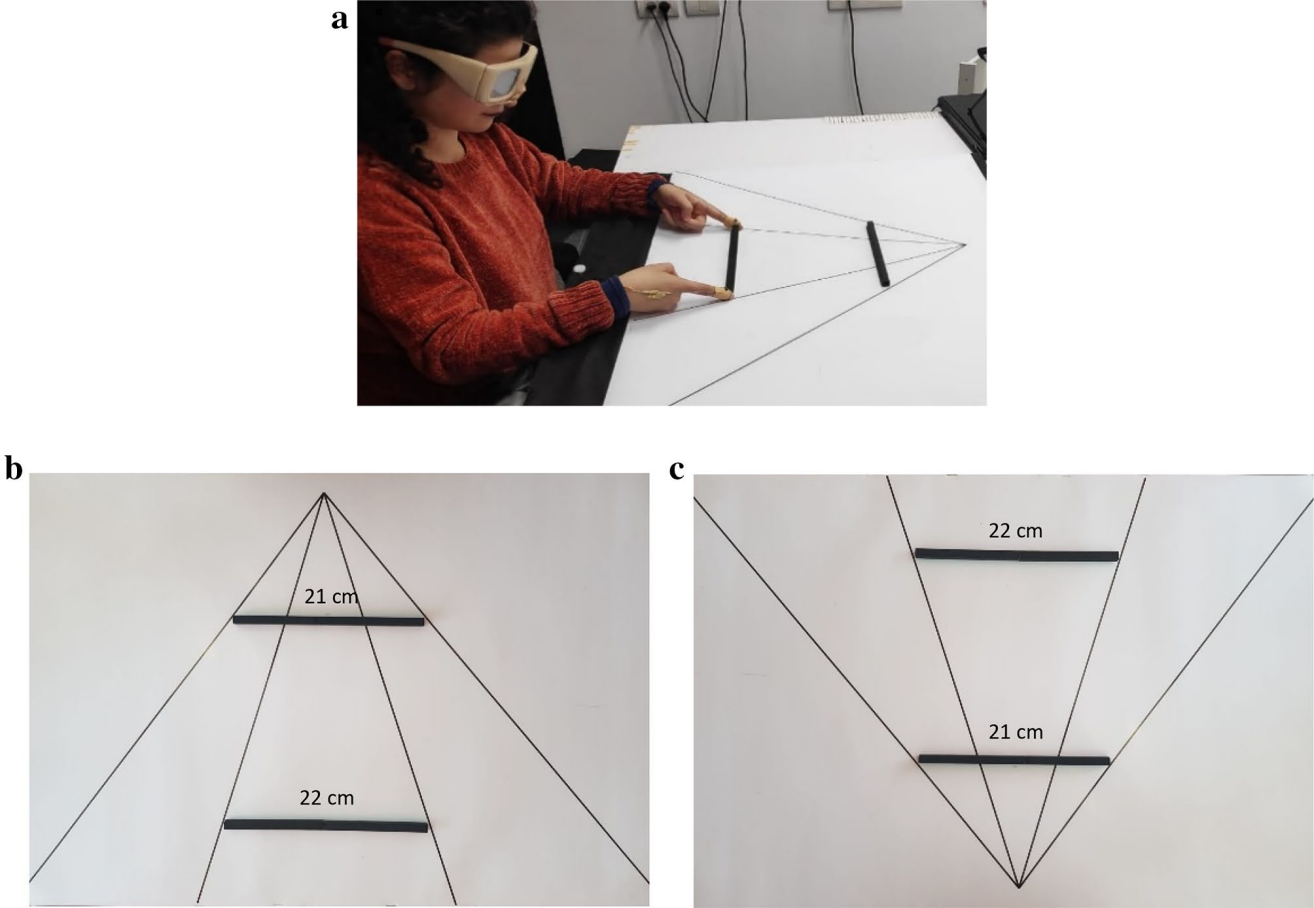
Stimuli and experimental design in Experiment 1. Participants grasped (**a**) or estimated the length of objects embedded in a standard (**b**) and inverted (**c**) configurations of the Ponzo illusion. Note that in both configurations, the physically shorter object appears longer.

Stimuli were three rectangular-shaped plastic rods (21 cm, 22 cm, 24.5 cm in length, 1 cm in width and depth, for the small, medium, and large object, respectively). Lengths were chosen based on pilot experimentation in which we measured the physical difference between the objects that could generate an illusory effect in the opposite direction. In the illusory background condition (grasping and manual estimations), two objects were placed on the upper and lower sides of a standard or inverted version of the Ponzo illusion (Fig. 1). The purpose of using the inverted configuration was to allow proper control for measuring the effect of the illusion for objects located at the same distance from the participant. Without the inverted configuration, the illusory effect would always be confounded with distance from the observer, and distance from the observer has been shown to have a significant effect on grip apertures^34^. Therefore, to focus on the effect of the illusion, it is important to compare grip apertures between objects located at the same distance from the participant.

In the control grasping condition, the objects were placed against a white background. The objects were always placed at fixed locations. The distance between the initial location of the fingers and the nearer object was 24 cm, and between the two objects was 22.5 cm. The participants’ viewing distance was approximately 40 cm for the nearer object.

### Procedure

The procedure was based on the procedure used by Ganel et al.^18^ and was adapted for bimanual grasping control. In Experiment 1a, participants grasped objects placed on the illusory background or the control, non-illusory background. The two conditions were performed in separate blocks, and block order was counterbalanced between participants. Before each trial, participants placed their index fingers around a small circular disk (2 cm in diameter), used as the starting point, while the goggles were set on their non-transparent state. In the illusory background condition, the participant was asked to lift the object she perceived as the shorter/longer in the pair using both her hands. A verbal command signaled the required judgment (“short”/”long”), and an additional tone signaled movement initiation. In the control grasping condition, the participant was asked to lift the closer/farther object according to the corresponding verbal command (“close”/”far”). Each trial began with the verbal command which was followed by the openning of the goggles that stayed in their transparent state for 5 s, allowing on-line vision of the display and objects during the trial. The go signal was presented 1 s after the opening of the goggles.

The design of Experiment 1b was similar to the one used in Experiment 1a (illusory background condition), except that now the participants estimated the sizes of objects by manually adjusting the distance between their fingers following the opening of the goggles. Perceptual estimations were performed on the lower right side of the tabletop. To allow equal haptic feedback to the one provided in the grasping condition, following each estimation trial the participants were asked to grasp and lift the target object using both hands. Following perceptual estimates, a “go” signal to grasp the object was presented 5 s after the opening of the goggles. The goggles remained open for additional 3 s to provide online vision during grasp.

In each illusory background condition (grasping; perceptual estimations), a total of sixty experimental trials were used. The standard configuration was presented for thirty consecutive trials, and the inverted configuration was presented in the remaining trials. Presentation order was counterbalanced between participants. Overall, there were 44 critical incongruent trials, in which the physically longer object (22 cm) was placed in the illusory background that induced an illusory effect in the opposite direction (22 trials in each illusory configuration). There were eight additional incongruent catch trials in which the physically shorter object was placed together with a noticeably larger object, 24.5 cm in length. In this configuration, the illusory background did not lead to reversal of the perceived size differences. In the eight remaining catch trials, the smaller and the larger objects were placed in illusory congruent locations. Trial order was pseudorandomized. In the control grasping condition, there were a total of thirty-two trials; in half of the trials, the 21 cm object was closer to the participant, and in the remaining trials the 22 cm object was closer (presentation order was counterbalanced). The 24.5 cm object was not presented in the control condition. Each condition started with a few practice trials.

### Data analysis

We recorded the 3D trajectories of the fingers in each trial. Movement onset was set as the point in time at which the aperture between two index fingers increased by more than 0.1 mm per frame for at least 75 ms. Movement offset was set as the point in time at which the aperture between the fingers changed less than 0.1 mm per frame for at least 125 ms (25 frames), but only after reaching the maximum grip aperture (MGA). The criteria used to detect movement onset and offset were based on the values used in a previous study from our lab in which we focused on unimanual grasps^35^. The values were slightly adjusted to allow effective identification of movement onset during bimanual grasping movements.

### Data analysis

For our primary analysis, we calculated the average MGA for each object size. The MGA provides reliable measure of the sensitivity of the grip aperture to object size^27,28^. To further analyze the movement trajectory, we divided the raw movement data to 11 equal intervals and calculated the average aperture in each interval (see Online Appendix 1). Detailed data for each of the participants in experiments 1 and 2 is provided in Online Appendix 2.

To avoid a potential confounding effect of obstacle avoidance when reaching out to grasp the distant object, we focused our main analysis only on near objects. Previous research suggests that physical stimuli in the target’s environment can be treated as obstacles and affect kinematic aspects of the action toward the target^37–40^. For example, studies have shown that stimuli in the target surroundings could lead to deviations in movement trajectories from their original location^39,40^. As can be seen in Figs. 1 and 3, in the current study the near objects could be treated as obstacles, and could potentially affect the apertures between the fingers in flight. This would not have been a major issue if the obstacles were identical in size. However, they are not; when reaching out to grasp the physically far small object, the obstacle is always large (22 cm in Experiment 1) and when reaching out to grasp the physically large far object, the obstacle is always small (21 cm in Experiment 1). This potential confound makes it difficult to interpret the results of grasping objects in a far location. We therefore do not include the objects in this location in our main analysis and focus on the near location. Grasping the near objects is free of potential influences of obstacle avoidance and provides valid measure of grasping performance of objects located in a fixed distance from the participants’ starting position.

Thus, we compared the MGA obtained in two separate configurations of the illusion (standard/ inverted). In Experiment 1, the critical comparison was between the 21 cm object presented on the inverted configuration of the illusion (and perceived in most trials as larger) and the 22 cm object on the standard configuration of the illusion (perceived in most trials as smaller). We note that previous studies argued that such a between-blocks analysis could potentially prevent unwarranted effects of an attentional mismatch^12^.

In addition, as in Ganel et al.’s^18^ study, in order to disentangle the effects of apparent and real size on grip apertures, we focused our analysis on the subset of trials in which the participants erroneously judged the object as shorter/longer due to the illusion. In Experiment 1a (illusory background condition), the participants' mean error rate for the incongruent trials was 80.9% (SD = 11.3, 35.2 trials on average). The error rate for near objects was 72% (SD = 15, 15.8 trials). The overall error rate in the perceptual estimation task was slightly larger overall (79%, SD = 16, 34.7 trials), as well as for the near objects (75%, SD = 17, 16.5). However, the difference in the overall error rate between the estimation and grasping conditions was not significant [*t*_(24)_ = 0.3, *p* = 0.7], as well as for the near objects [*t*_(24)_ = 0.3, *p* = 0.6].

### Results and discussion

**Figure 2:**
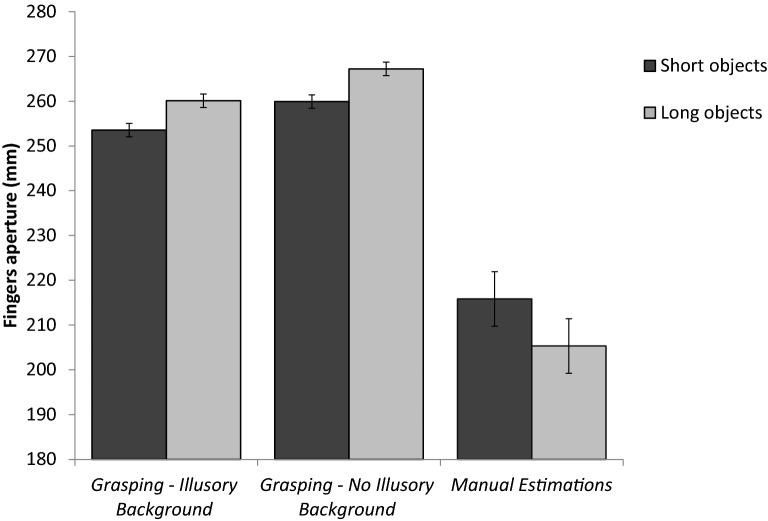
The results of experiments 1a and 1b. For grasping, apertures reflected the actual size differences between the objects, even in trials in which participants made erroneous judgments of size. In contrast, manual estimations reflected the perceived, illusory size difference between the objects. Error bars represent confidence intervals in repeated measures ANOVAs^55^.

The main results are presented in Fig. 2. As can be seen in the figure, a dissociable pattern of performance was obtained for grasping grip apertures and for perceptual estimates of size. For grasping (Experiment 1a), the scaling of apertures reflected the actual size differences between the objects rather than their apparent sizes [*t*_(13)_ = 4.2, *p* = 0.001]. The MGA was larger for an object that was perceived as smaller (253, 260 mm, for the short and large objects, respectively). In sharp contrast to grasping, manual estimations (Experiment 1b) went in the same direction as the overt perceptual judgments and were biased by the illusion. Therefore, the distance between the fingers during perceptual estimations reflected the illusory size difference between the objects [215, 205 mm, for the short and large objects, respectively, *t*_(12)_ = 2.6, *p* = 0.02]. A mixed model ANOVA was conducted on grip apertures with experiment as a between-subjects independent variable and size as a within-subject factor. There was a significant main effect for experiment [*F*_(1*,*25)_ = 94*.*6*, p* < 0*.*001*, η*_*p*_^2^ = 0*.*80], with larger apertures for grasping (measured at MGA), compared to manual estimations (257, 210 mm, respectively). The main effect of size was not significant [*F*_(1,25)_ = 0*.*92*, p* = 0*.*3]. Importantly, the interaction between physical size and experiment was significant [*F*_(1*,*25)_ = 17*.*2,* p* < 0*.*001*, η*_*p*_^2^ = 0*.*40].

The pattern of results in the control grasping condition in Experiment 1a was similar to that found in the main grasping condition with the illusory background. The MGA was smaller for the 21 cm object compared to the 22 cm object [260, 267 mm, *t*_(13)_ = 6.0-, *p* < 0.001]. To look for potential differences between the illusion and control grasping condition, a repeated-measures ANOVA test with condition and size as within-subject variables was conducted on the MGA data. The test showed a main effect for condition [*F*_(1,13)_ = 13.5,* p* = 0*.*003,* η*_*p*_^2^ = 0*.*50], with larger apertures in the control condition compared to the illusion condition (263, 256 mm, respectively). The main effect of size was also significant [*F*_(1,13)_ = 51*.*7*, p* < 0*.*001*, η*_*p*_^2^ = 0*.*80]. The difference in overall MGA between the control condition and the illusory condition was not predicted and we do not have a solid account for this effect. More importantly, however, there was no interaction between condition and size [*F*_(1*,*13)_ = 0.13*, p* = 0*.*7], indicating similar sensitivity to physical size differences in the two conditions. An analysis of the grip apertures across the movement trajectory in Experiment 1a is presented in Online Appendix 1. The pattern of results was similar to that obtained for the MGA data. Again, grip apertures reflected the real size differences between the objects. Mean MGAs and grip apertures in the manual estimation condition across the different distances from the target are presented in Table 1.

**Table 1:** Mean MGAs and perceptual estimates (in mm) for the near and far objects in experiments 1–2. standard deviations are in brackets.

Object location		*Near*		*Far*		*Overall*	
Object size		*Short*	*Long*	*Short*	*Long*	*Short*	*Long*
Experiment 1	Grasping	253 (12)	260 (12)	264 (24)	268 (18)	258 (17)	264 (14)
	Grasping control	260 (9)	267 (10)	266 (18)	277 (19)	263 (13)	272 (14)
	Manuel estimations	215 (14)	205 (15)	220 (20)	199 (16)	218 (16)	202 (14)
Experiment 2	Grasping	219 (9)	224 (10)	236 (14)	239 (16)	228 (11)	231 (11)
	Manuel estimations	186 (24)	171 (19)	187 (23)	173 (25)	186 (23)	172 (21)

The results of experiments 1a and 1b extend and replicate previous findings obtained in the domain of unimanual control^18^. While bimanual estimations reflected the illusory size difference between the objects, bimanual grasping apertures reflected their actual difference in size. In Experiment 2, we tested whether this pattern of results extends to a different illusion of size, the Wundt–Jastrow illusion.

## Experiment 2

Fourteen participants (4 males, average age = 25.5, SD = 2.3), who did not participate in Experiment 1, participated in Experiment 2a (bimanual grasping condition), and ten other participants, who did not participate in Experiment 1, (1 male, average age = 23.6, SD = 1.7) participated in Experiment 2b (bimanual estimation condition). The results of two participants were excluded from the analysis. One participant (Experiment 2a), who did not follow the instructions, and another participant (Experiment 2b) due to a failure to capture her movements.

### Stimuli and design

**Figure 3:**
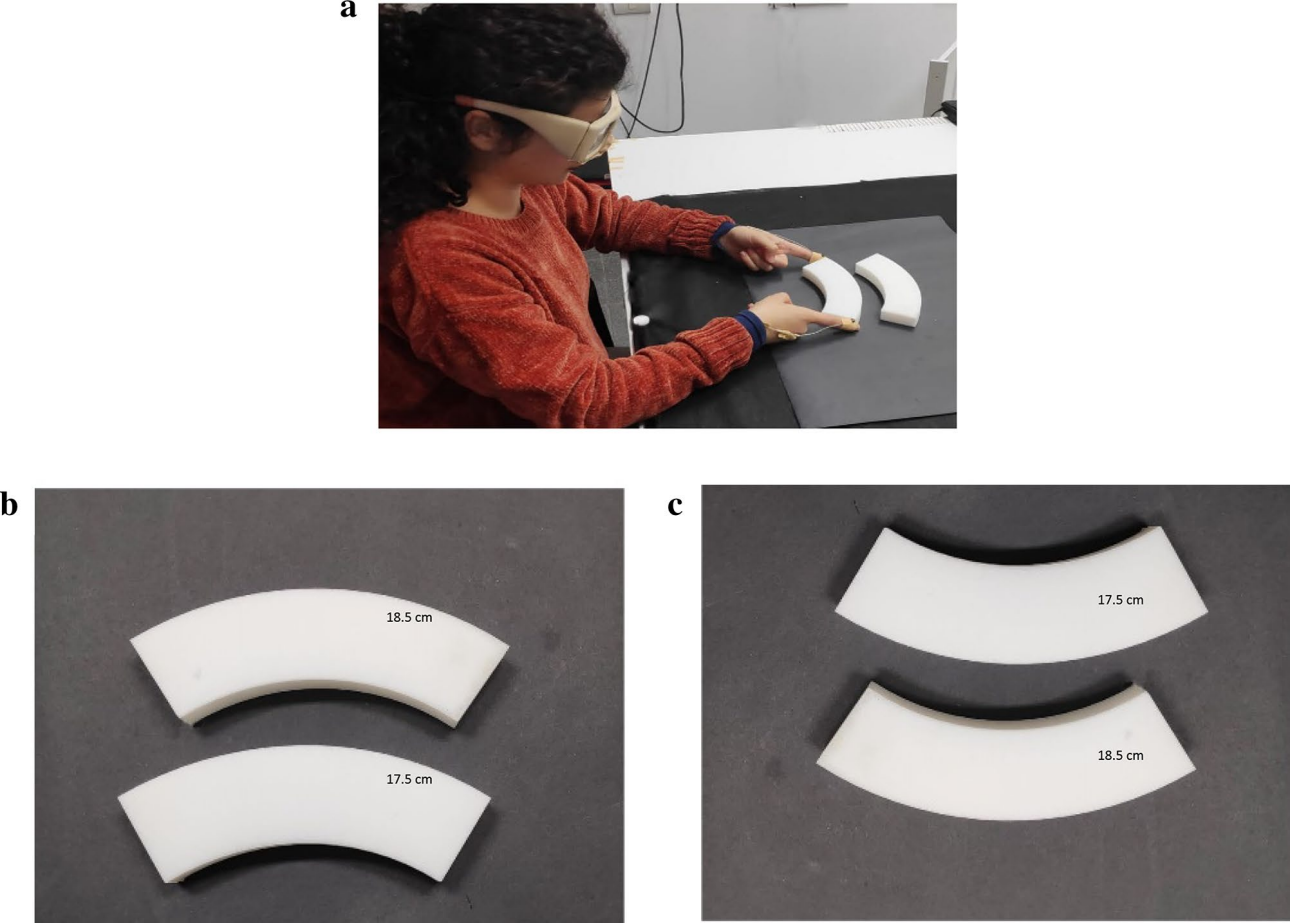
Stimuli and experimental design used in Experiment 2. Participants either grasped (**a**) or estimated the horizontal length of the standard (**b**) and the inverted (**c**) 3D configurations of the Wundt–Jastrow illusion. The physically shorter object appeared bigger in the majority of the trials.

The overall design was similar to the one used in Experiment 1, except that now participants grasped different sized fan-shaped objects that constitute the Wundt–Jastrow Illusion. In the classic (2d) version of this illusion, the two fans are presented next to one other so that the inner circle of one object is vertical to the exterior circle of the other. This spatial organization leads to the perception that the inner fan is considerably bigger than the outer fan^41,42^ (also see^43^ on the similarities between the Ponzo and the Wundt–Jastrow illusions). In the modified 3D version used in Experiment 2, the two fans were physically different in size. For the critical incongruent trials, the physically shorter object was placed in the inner position and appeared (in most trials) as larger (Fig. 3a,b). Three different-sized objects were used; all objects were 4 cm in width and 2 cm in depth. The lengths between the top edges were 17.5, 18.5 and 21.5 cm for the small, medium, and big objects, respectively (the radiuses between the top edges to a reference point located 11 cm below its center point were 68, 70, 88 degrees, respectively). The largest object was used for the incongruent catch trials. The distance between the fingers' starting position to the nearest object was the same as in Experiment 1. The horizontal distance between the two target objects was 3 cm. As in Experiment 1 (illusory background conditions), a total of sixty experimental trials were presented, and each version of the illusion (standard/ inverted) was presented in 30 consecutive trials (mini-block order was counterbalanced between participants). The participants were asked to lift one of the objects by their external edges (Fig. 3a). Overall rates of susceptibility to the illusion in the grasping experiment were relatively high (90.1%, SD = 7, 40 trials on average). The error rate for near objects was 85% (SD = 11, 18.7 trials). The overall error rate for the perceptual estimations was 81% (SD = 14, 35.6 trials) and 76%, for the near objects (SD = 18, 16.7 trials). The differences between grasping and manual estimations in terms of overall error rate [*t*_(20)_ = 1.9, *p* = 0.06] as well as for near objects [*t*_(20)_ = 1.5, *p* = 0.14] did not reach statistical significance.

### Results and discussion

**Figure 4:**
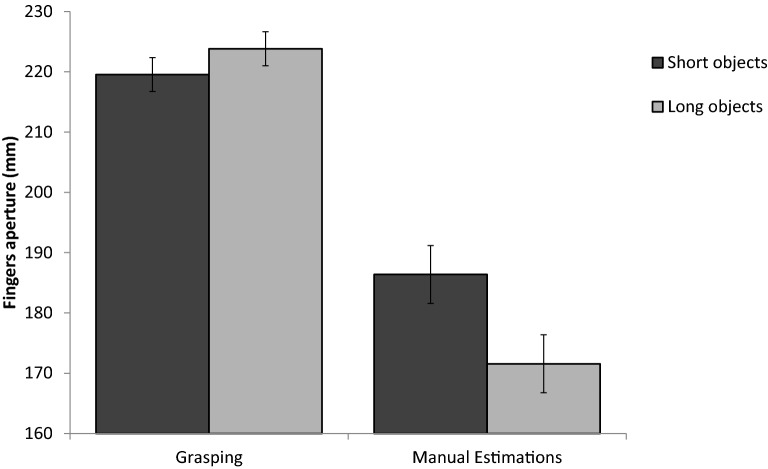
The results of experiments 2a and 2b. MGAs during bimanual grasping reflected the actual size differences between the objects, even in the trials in which participants made erroneous perceptual judgments. In contrast, manual estimations reflected the illusory, perceived size differences between the objects. Error bars represent confidence intervals in repeated measures ANOVAs^55^.

The main results are presented in Fig. 4. The findings closely match those obtained in Experiment 1. Bimanual grasping apertures and bimanual perceptual estimations for the critical trials in which participants erroneously perceived the larger object in the pair as smaller showed an opposite pattern of sensitivity to real size. For grasping, MGAs were larger for the object that was perceived as smaller [219, 224 mm, for the short and large objects, respectively, *t*_(12)_ = 2.3, *p* = 0.03]. In contrast, in the perceptual estimation task, finger apertures reflected the illusory size differences between the objects [186, 171 mm, for the short and large objects, respectively, *t*_(8)_ = 3.9, *p* = 0.004]. These findings document a pattern of a double dissociation between grasping and perceptual estimates in terms of their sensitivity to real and to illusory size. A mixed-model ANOVA with experiment as a between-subjects independent variable and size as a within-subject factor was conducted on the aperture data. There was a main effect for condition [*F*_(1*,*20)_ = 41*, p* < 0*.*001*, η*_*p*_^2^ = 0*.*67]; grip apertures were larger for grasping compared to manual estimations task (221, 179 mm, respectively). The main effect of size was also significant [*F*_(1*,*20)_ = 7.7*, p* = 0*.*01*, η*_*p*_^2^ = 0.28]. More importantly, there was a significant interaction between condition and size [*F*_(1*,*20)_ = 25*.*6*, p* < 0*.*001*, η*_*p*_^2^ = 0*.*56], which reflected the opposite effects of physical and illusory size differences on grasping and on perceptual estimates. An additional analysis of the movement trajectory in the bimanual grasping condition is presented in Online Appendix 1. As in Experiment 1, the grip apertures reflected the actual size differences between the objects. Mean grip apertures for the remaining conditions are presented in Table 1.

## General discussion

The results show that the double dissociation between grasping and perceptual judgments, previously documented for unimanual grasping, extends to bimanual grasping control. A similar pattern of results was obtained in experiments 1 and 2. In particular, grip apertures during grasping reflected the real size differences between the objects. This pattern of results was found in the same trials in which the participants erroneously perceived the larger object in the pair as the smaller one. Therefore, in the context of both illusions, grip apertures during bimanual grasping were accurately scaled to the physical size differences between the objects. Furthermore, when participants adjusted the distance between their fingers to match the perceived size of the objects, their perceptual estimates reflected the illusory rather than the physical differences in size. These findings are in line with the results of previous studies conducted on unimanual motor control^5,9,18,44^ and suggest that bimanual and unimanual grasping control may rely on shared visuomotor representations based on real rather than on relative world metrics.

Our findings are also consistent with a recent study from our lab in which we tested the adherence of bimanual grasping and bimanual estimations to Weber’s law. In this study^31^, participants were asked to either grasp or estimate different-sized objects using both their hands. In line with findings from unimanual movement control, JNDs measured for estimation increased with size, adhering to Weber’s law. However, JNDs during grasping did not increase with size, in violation of Weber’s law. These results were interpreted based on the idea that the control of visually guided action relies on a more analytic processing style compared to perceptual processing^31,45^. The current study extends these findings by showing that bimanual grasping movements are refractory to relative, task-irrelevant information. These results suggest that the division of labor between the system that mediates the control of action and the system that mediates perceptual processing generalizes to bimanual motor control.

In the current set of experiments, a closed-loop design, in which vision is allowed throughout the entire trial was used both in the grasping and in the manual estimation conditions. This was done to ensure similar visual feedback in the two conditions. We note that there are pros and cons for using closed-loop and open-loop designs in grasping experiments. On the one hand, open-loop designs prevent the participants from using feedback from their fingers during grasp^46^. But on the other hand, they make the task of grasping less like what happens in the real world^19,47^. Therefore, it is difficult to tell if any illusory effect during open-loop grasping is due to the prevention of visual feedback from the fingers and objects or due to the inefficiency of the task in hand^47^. In the current study we used a closed-loop design, a design that we typically use in other studies in our lab^35,48^. Therefore, the current findings reflect performance during grasping and perceptual estimations when full vision is allowed throughout the entire trial.

Another issue related to the current experimental design is the requirement from participants to grasp the objects using their both hands from its horizontal plane (see Figs. 1 and 3). We note that such a requirement may affect the ecological validity of the design because unless explicitly asked for, participants may have grasped the objects along their vertical plane using unimanual grasps rather than along the horizontal plane that requires picking up the objects with both hands. This issue could be attenuated if we would have used a set of wider objects, which would make it difficult to grasp the objects with one hand and would limit grasping choices to bimanual grasping. Yet, using wider objects would also limit their potential presentation in the context of illusions, which is why we chose the current set of objects for the two experiments. It could be argued, however, that the objects that we used can afford unimanual grasps, which may in turn lead to additional visual processing and perhaps to performing the movements in an unusual manner. Yet, we also note that previous research showed that unusual and awkward grasps are more likely to be affected by illusions^19,30^. Therefore, a potential effect of unusual grasp would be in a direction opposite to the direction of the current findings. Therefore, the current results support the idea that despite the potential effect of unusual grasp affordance or requirements, grasping movements were still performed efficiently, and could escape potential influences of illusory distortions.

There is a current debate among investigators as to the extent to which (some) visual illusions affect action control. For instance, some previous studies argued that the resistance of reach to grasp movements to the Ebbinghaus Illusion could be accounted for by methodological issues^15^. Unlike in the case of the Ebbinghaus illusion, previous studies that used the Ponzo illusion showed a more consistent pattern of a dissociation between action and perception in terms of their resistance to the illusory effect^9,18–20^. The current design allowed us to test the relative contribution of real and of illusory size differences to action and to perception^18^. Our results provide support for the idea that bimanual grasping can effectively resist illusory context.

To the best of our knowledge, the present study was the first to test the potential effect of the Wundt–Jastrow illusion on grasping movements (Experiment 2). One advantage of using this modified version of the illusion in grasping experiments (compared to other commonly used illusions) is the lack of pictorial background. According to previous studies, the presence of illusory background could be treated as an obstacle and affect the in-flight scaling of the fingers prior to grasp^7,49^ (but see^50^). It is reasonable to assume that in a similar fashion to bimanual grasping, unimanual grasping movements will resist the Wundt–Jastrow Illusion. This conclusion, however, awaits further experimentation.

Previous studies proposed that bimanual grasping control relies on a different, although partly overlapping neural circuitry, compared to unimanual grasping, and involves the encoding of different aspects of the visual scene^22,23^. The findings of the current study highlight the functional similarity between bimanual and unimanual grasping. Nevertheless, it is still possible that bimanual and unimanual motor control differ with respect to their vulnerability to perceptual illusions. Indeed, previous research has shown that type of interaction with the object could modulate vulnerability to perceptual effects^19,35,51–54^. Future studies should explore potential differences between unimanual and bimanual control in terms of the computations that mediate these actions.

To summarize, the present findings extend the previously established dissociation between action and perception in the context of visual illusions to bimanual grasping control. The findings suggest that despite potential differences between unimanual and bimanual control, both types of actions share a common representation of the visual environment. This representation differs from the one that mediates the visual perception of objects and is more closely tuned to their actual properties.

Statement regarding experimental protocols.

All experimental protocols in experiments 1 and 2 was approved by the Human Subjects Research Committee at Ben-Gurion University of the Negev (submission #1257). The study adhered to the ethical standards of the Declaration of Helsinki. Informed consent was obtained from all participants of experiment 1 and 2.

## Supplementary information

Supplementary information 1.

Supplementary information 2.

## References

[1] Goodale MA, Milner AD (2018). Two visual pathways—where have they taken us and where will they lead in future?. Cortex J. Devoted Study Nerv. Syst. Behav..

[2] Kravitz DJ, Saleem KS, Baker CI, Mishkin M (2011). A new neural framework for visuospatial processing. Nat. Rev. Neurosci..

[3] Milner AD, Goodale MA (2008). Two visual systems re-viewed. Neuropsychologia.

[4] Aglioti S, DeSouza JFX, Goodale MA (1995). Size-contrast illusions deceive the eye but not the hand. Curr. Biol..

[5] Carey DP (2001). Do action systems resist visual illusions?. Trends Cogn. Sci..

[6] Goodale MA (2014). How (and why) the visual control of action differs from visual perception. Proc. R. Soc. B Biol. Sci..

[7] Haffenden AM, Schiff KC, Goodale MA (2001). The dissociation between perception and action in the Ebbinghaus illusion: nonillusory effects of pictorial cues on grasp. Curr. Biol..

[8] Haffenden AM, Goodale MA (1998). The effect of pictorial illusion on prehension and perception. J. Cogn. Neurosci..

[9] Whitwell RL, Buckingham G, Enns JT, Chouinard PA, Goodale MA (2016). Rapid decrement in the effects of the Ponzo display dissociates action and perception. Psychon. Bull. Rev..

[10] Kopiske KK, Cesanek E, Campagnoli C, Domini F (2017). Adaptation effects in grasping the Müller–Lyer illusion. Vision Res..

[11] Smeets JBJ, Brenner E (2006). 10 years of illusions. J. Exp. Psychol. Hum. Percept. Perform..

[12] Franz VH, Gegenfurtner KR, Bülthoff HH, Fahle M (2000). Grasping visual illusions: no evidence for a dissociation between perception and action. Psychol. Sci..

[13] Franz VH (2003). Manual size estimation: a neuropsychological measure of perception?. Exp. Brain Res..

[14] Stöttinger E, Soder K, Pfusterschmied J, Wagner H, Perner J (2010). Division of labour within the visual system: fact or fiction? Which kind of evidence is appropriate to clarify this debate?. Exp. Brain Res..

[15] Franz VH, Gegenfurtner KR (2008). Grasping visual illusions: Consistent data and no dissociation. Cogn. Neuropsychol..

[16] Kopiske KK, Bruno N, Hesse C, Schenk T, Franz VH (2016). The functional subdivision of the visual brain: Is there a real illusion effect on action? A multi-lab replication study. Cortex.

[17] Whitwell RL, Goodale MA (2017). Real and illusory issues in the illusion debate (Why two things are sometimes better than one): commentary on Kopiske et al. (2016). Cortex J. Devoted Study Nerv. Syst. Behav..

[18] Ganel T, Tanzer M, Goodale MA (2008). A double dissociation between action and perception in the context of visual illusions: opposite effects of real and illusory size. Psychol. Sci..

[19] Gonzalez CLR, Ganel T, Whitwell RL, Morrissey B, Goodale MA (2008). Practice makes perfect, but only with the right hand: sensitivity to perceptual illusions with awkward grasps decreases with practice in the right but not the left hand. Neuropsychologia.

[20] Jackson SR, Shaw A (2000). The Ponzo illusion affects grip-force but not grip-aperture scaling during prehension movements. J. Exp. Psychol. Hum. Percept. Perform..

[21] Cesanek E, Campagnoli C, Taylor JA, Domini F (2018). Does visuomotor adaptation contribute to illusion-resistant grasping?. Psychon. Bull. Rev..

[22] Le A, Vesia M, Yan X, Niemeier M, Crawford JD (2014). The right anterior intraparietal sulcus is critical for bimanual grasping: a TMS study. Cereb. Cortex.

[23] Le A, Niemeier M (2013). Left visual field preference for a bimanual grasping task with ecologically valid object sizes. Exp. Brain Res..

[24] Le A, Niemeier M (2013). A right hemisphere dominance for bimanual grasps. Exp. Brain Res..

[25] Gonzalez CLR, Ganel T, Goodale MA (2006). Hemispheric specialization for the visual control of action is independent of handedness. J. Neurophysiol..

[26] Radoeva PD, Cohen JD, Corballis PM, Lukovits TG, Koleva SG (2005). Hemispheric asymmetry in a dissociation between the visuomotor and visuoperceptual streams. Neuropsychologia.

[27] Corballis PM (2003). Visuospatial processing and the right-hemisphere interpreter. Brain Cogn..

[28] Kelley WM (1998). Hemispheric specialization in human dorsal frontal cortex and medial temporal lobe for verbal and nonverbal memory encoding. Neuron.

[29] Fisk JD, Goodale MA (1988). The effects of unilateral brain damage on visually guided reaching: hemispheric differences in the nature of the deficit. Exp. Brain Res..

[30] Vishton PM, Fabre E (2003). Effects of the Ebbinghaus illusion on different behaviors: one-and two-handed grasping; one- and two-handed manual estimation; metric and comparative judgement. Spat. Vis..

[31] Ganel T, Namdar G, Mirsky A (2017). Bimanual grasping does not adhere to Weber’s law. Sci. Rep..

[32] Dewar MT, Carey DP (2006). Visuomotor ‘immunity’ to perceptual illusion: a mismatch of attentional demands cannot explain the perception–action dissociation. Neuropsychologia.

[33] Foster RM, Kleinholdermann U, Leifheit S, Franz VH (2012). Does bimanual grasping of the Müller–Lyer illusion provide evidence for a functional segregation of dorsal and ventral streams?. Neuropsychologia.

[34] Jakobson LS, Goodale MA (1991). Factors affecting higher-order movement planning: a kinematic analysis of human prehension. Exp. Brain Res..

[35] Ozana A, Ganel T (2019). Weber’s law in 2D and 3D grasping. Psychol. Res..

[36] Jeannerod M (1986). The formation of finger grip during prehension. A cortically mediated visuomotor pattern. Behav. Brain Res..

[37] Dean J, Brüwer M (1994). Control of human arm movements in two dimensions: paths and joint control in avoiding simple linear obstacles. Exp. Brain Res..

[38] Tresilian JR (1998). Attention in action or obstruction of movement? A kinematic analysis of avoidance behavior in prehension. Exp. Brain Res..

[39] Garzorz IT, Knorr AG, Gilster R, Deubel H (2018). The influence of obstacles on grasp planning. Exp. Brain Res..

[40] Chapman CS, Goodale MA (2008). Missing in action: the effect of obstacle position and size on avoidance while reaching. Exp. Brain Res..

[41] Coren S, Girgus JS (1978). Seeing is Deceiving: The Psychology of Visual Illusions.

[42] Robinson JO (2013). The Psychology of Visual Illusion.

[43] Pick DF, Pierce KA (1993). Theoretical parallels between the Ponzo illusion and the Wundt–Jastrow illusion. Percept. Mot. Skills.

[44] Stöttinger E, Perner J (2006). Dissociating size representation for action and for conscious judgment: grasping visual illusions without apparent obstacles. Conscious. Cogn..

[45] Ganel T, Chajut E, Algom D (2008). Visual coding for action violates fundamental psychophysical principles. Curr. Biol..

[46] Glover S, Dixon P (2001). The role of vision in the on-line correction of illusion effects on action. Can. J. Exp. Psychol. Can. Psychol. Exp..

[47] Whitwell RL, Goodale MA, Merritt KE, Enns JT (2018). The Sander parallelogram illusion dissociates action and perception despite control for the litany of past confounds. Cortex.

[48] Ozana A, Ganel T (2018). Dissociable effects of irrelevant context on 2D and 3D grasping. Atten. Percept. Psychophys..

[49] Haffenden AM, Goodale MA (2000). Independent effects of pictorial displays on perception and action. Vision Res..

[50] Franz VH, Bülthoff HH, Fahle M (2003). Grasp effects of the Ebbinghaus illusion: obstacle avoidance is not the explanation. Exp. Brain Res..

[51] Goodale MA, Jakobson LS, Keillor JM (1994). Differences in the visual control of pantomimed and natural grasping movements. Neuropsychologia.

[52] Holmes SA, Heath M (2013). Goal-directed grasping: the dimensional properties of an object influence the nature of the visual information mediating aperture shaping. Brain Cogn..

[53] Hu Y, Goodale MA (2000). Grasping after a delay shifts size-scaling from absolute to relative metrics. J. Cogn. Neurosci..

[54] Westwood DA, Goodale MA (2003). Perceptual illusion and the real-time control of action. Spat. Vis..

[55] Jarmasz J, Hollands JG (2009). Confidence intervals in repeated-measures designs: the number of observations principle. Can. J. Exp. Psychol. Can. Psychol. Exp..

