# Supplementary material for: A double dissociation between action and perception in bimanual grasping: evidence from the Ponzo and the Wundt–Jastrow illusions
Source: Sci Rep. 2020 Sep 4;10:14665. doi: 10.1038/s41598-020-71734-z (PMC7473850; doi:10.1038/s41598-020-71734-z)
Supplement: Supplementary file 1 — Supplementary information 1. [file 41598_2020_71734_MOESM1_ESM.docx]

Supplementary material

A double dissociation between action and perception in bimanual grasping: Evidence from the Ponzo and the Wundt-Jastrow illusions

Aviad Ozana and Tzvi Ganel

Department of Psychology, Ben-Gurion University of the Negev, Beer-Sheva 8410500, Israel.

Corresponding author:

Tzvi Ganel, Ph.D.

Department of Psychology

Ben-Gurion University of the Negev

Beer-Sheva 8410500

ISRAEL

Phone: +972-8-6428515

Fax: +972-8-6428348

Email: [tganel@bgu.ac.il](mailto:tganel@bgu.ac.il)

Running title: Grasping illusions

Keywords: Perception and action, grasping, Ponzo illusion, Wundt-Jastrow illusion, object perception.

# Appendix 1: Experiments 1-2: Additional kinematic measures

**Figure S1:** Average grip apertures across the normalized movement in Experiment 1a (illusory-background condition, near objects). Grasping trajectories reflected the actual size differences between the objects [*F_(1,13)_ =5.7, p =.03, η_p_^2^=0.30*], in trials in which participants made erroneous judgments of size. Error bars represent confidence intervals in repeated measures ANOVAs.

**Figure S2:** Average grip apertures across the normalized movement in Experiment 2 (near objects). The grip aperture reflected the actual size differences between the objects [*F_(1,12)_ =4.7, p =.05, η_p_^2^=0.28*], in trials in which participants made erroneous judgments of size. Error bars represent confidence intervals in repeated measures ANOVAs.
